# Comparative transcriptomics reveals context- and strain-specific regulatory programmes of Agrobacterium during plant colonization

**DOI:** 10.1099/mgen.0.001485

**Published:** 2025-08-22

**Authors:** Yu Wu, Hsin-Yi Chang, Chih-Hang Wu, Erh-Min Lai, Chih-Horng Kuo

**Affiliations:** 1 Institute of Plant and Microbial Biology, Academia Sinica, Taipei 115201, Taiwan, ROC; 2 Molecular and Biological Agricultural Sciences Program, Taiwan International Graduate Program, National Chung-Hsing University and Academia Sinica, Taipei 115201, Taiwan, ROC; 3 Graduate Institute of Biotechnology, National Chung-Hsing University, Taichung 402202, Taiwan, ROC; 4 Biotechnology Center, National Chung-Hsing University, Taichung 402202, Taiwan, ROC

**Keywords:** *Agrobacterium*, plant pathogen, RNA-Seq, transcriptomics, transformation, virulence

## Abstract

*Agrobacterium* is a genus of plant-associated bacteria capable of transferring DNA into host genomes to induce tumourigenesis. The process has been primarily studied in a few model strains, particularly C58, and developed into *Agrobacterium*-mediated transformation for genetic manipulation. However, the diversity of wild-type strains and their context-specific regulatory responses remains poorly characterized. Here, we evaluated five wild-type strains and identified 1D1108 as superior in tumourigenesis on legumes and transient transformation in *Nicotiana benthamiana*. Under *in vitro* virulence induction with acetosyringone, we identified 126 differentially expressed genes (DEGs) in 1D1108. Although the number of DEGs was comparable to those in C58 and the legume isolate 1D1609 under the same condition, only 22 DEGs, primarily within the *vir* regulon, were conserved, indicating extensive divergence among these *Agrobacterium* strains. Leaf infiltration of *N. benthamiana* revealed 1,134 DEGs specifically regulated *in planta* for 1D1108. These included genes involved in attachment, virulence regulation, type IV pilus, succinoglycan biosynthesis and diverse nutrient transporters, providing new evidence on expression regulation during colonization. Comparative analyses of *in planta* transcriptomes with C58 and *Pseudomonas syringae* DC3000 revealed distinct secretion systems required for pathogenesis, namely, type IV for *Agrobacterium* and type III for *Pseudomonas*, and only ~5–19% of DEGs were conserved. These limited transcriptomic overlaps underscore the importance of studying gene expression in strains and conditions directly relevant to the biological context, rather than relying on model systems. Together, this work reveals how environmental and host-associated cues shape transcriptional responses in plant-associated bacteria.

**Impact Statement**

*Agrobacterium* is a key tool for plant genetic engineering, yet much of our knowledge about its biology comes from a few strains studied under artificial conditions. This work combines tumour formation assays on legumes and transient transformation in *Nicotiana benthamiana* with transcriptomic analyses to explore how diverse *Agrobacterium* strains, particularly the high-performing wild-type strain 1D1108, respond to host and environmental cues. By comparing gene expression under *in vitro* and *in planta* conditions, we found that the bacterial response within plant tissue is more complex than previously appreciated. Cross-strain comparisons further revealed limited conservation in gene expression regulation, even under similar conditions. Comparative analysis with *Pseudomonas syringae* revealed the activation of distinct secretion systems for pathogenesis and differences in regulatory programmes used during plant colonization. These findings underscore the importance of studying bacteria in biologically relevant contexts and highlight the limitations of relying solely on model strains to infer regulatory responses.

## Data Summary

All Illumina RNA-Seq datasets are available in the National Center for Biotechnology Information under BioProject accession PRJNA1111437.

## Introduction

The genus *Agrobacterium* contains a group of soil bacteria associated with plants and is notable for its capacity to cause crown gall or hairy root diseases [1,2]. The phytopathogenicity of these bacteria depends on oncogenic plasmids such as tumour-inducing plasmids (pTi) or root-inducing plasmids (pRi) [3,4]. During the infection process, a specific segment of transfer DNA (T-DNA) located on the oncogenic plasmid is processed and transported into the host cells via the type IV secretion system (T4SS) encoded on the same plasmid [5,6]. The T-DNA can be integrated into the plant nuclear genome, leading to expression of T-DNA genes for the biosynthesis of plant hormones and opines. The transformation not only results in abnormal proliferation of plant cells but also converts the plant cells into food factories for the invading bacteria.

This unique interkingdom DNA transfer has been exploited by biologists to develop *Agrobacterium*-mediated transformation (AMT) as a powerful tool for genetic manipulation of plants [5,7]. Compared to the gene gun method, which uses microparticle bombardment, AMT has several key advantages including low cost, ease of use and minimal damages to the target genome. Moreover, in addition to stable transformation, AMT can also be used for transient expression of genes encoded by T-DNA without integration. Therefore, AMT offers flexibility for the study of gene functions in plants and the generation of genetically modified organisms for biotechnology applications.

To develop AMT for genetic engineering, many studies have been devoted to investigating the molecular mechanisms and genes involved [5,6]. The infection process starts from the attachment of bacterial cells to the host, which involves beta-1,2 glucan production by ChvB and transport by ChvA [8,9]. Effective infection involves recognition of plant wounding signals, including acidity at the pH range of 5.5–6.0 commonly found in plant apoplast [10], as well as monosaccharides and phenolic compounds such as acetosyringone (AS) [11]. These signals can be sensed by bacterial two-component systems, such as those encoded by *chvG*/*chvI* for pH change [12] and *virA*/*virG* for phenolics [13], leading to the expression of virulence (*vir*) genes located on oncogenic plasmids. Subsequently, T-DNA is processed and transferred into the host cells by various Vir proteins, including VirB1-11 and VirD4, that together constitute a functional T4SS [14].

Despite the advancements, two critical knowledge gaps remained regarding agrobacterial gene expression regulation. First, the genus *Agrobacterium* harbours extensive biological diversity, but studies on virulence have focused on only a few strains. To date, more than 20 genomospecies have been defined based on genomic divergence [6]. Strains belonging to the same genomospecies may differ by up to 15% of their gene content, while strains belonging to different genomospecies may differ by more than 20% in gene content [15,17]. In addition to the diversity of chromosomal genes, pTi can be classified into at least 11 types [3,4, 15]. Importantly, the genetic diversity is likely linked to phenotypic diversity such as host range and transformation efficiencies [18]. To date, most studies on agrobacterial virulence have focused on strain C58 belonging to genomospecies 8, which is also the wild-type progenitor of nearly all disarmed strains commonly used for AMT such as GV3101 and EHA105 [5,7]. Second, for transcriptomics investigation, most previous studies not only focused on C58 but also mainly examined *in vitro* conditions [19,25]. Two exceptions include one that compared C58 to a genomospecies 7 strain 1D1609 for their responses to *in vitro* AS treatment by RNA-Seq [17] and one that investigated C58 gene expression inside *Arabidopsis thaliana* tumours by microarrays [26].

To bridge the aforementioned gaps and to expand our understanding of *Agrobacterium* biology, we conducted tumourigenesis and transformation assays of five wild-type *Agrobacterium* strains (Table 1). These strains differ from C58 in their host range according to artificial inoculation experiments [18]. Also, our genomic characterization revealed their divergence in genomospecies assignment, gene content and pTi type [15,16]. We discovered that strain 1D1108 consistently exhibited high efficiencies of inducing tumour formation in kidney bean and soybean, as well as a high transient transformation efficiency in *Nicotiana benthamiana*. Therefore, we conducted RNA-Seq experiments to investigate 1D1108’s transcriptomic responses to acidic environment and AS treatment under *in vitro* conditions, as well as under an *in planta* condition following infiltration into *N. benthamiana* leaves. Through these experiments, together with comparisons with previous *Agrobacterium* transcriptomics results [17,19, 26] and *in planta* transcriptomics of *Pseudomonas syringae* [27,28], we identified the commonalities and specificity in gene expression regulation among diverse plant pathogens in response to different environmental cues.

**Table 1.** The *Agrobacterium* strains used in this study. The genomospecies and pTi type assignment are based on [3] (DOI: 10.1126/science.aba5256)

Strain	Natural host	NCBI accession	Genomo -species	pTi type	Opine type	Reference
1D1108	*Euonymus*	GCA_003666425.1	G1	I.b	Nopaline	Wroblewski et al. (2005); DOI: 10.1111/j.1467–7652.2005.00123.x
1D1460	Raspberry vine	GCA_003666445.1	G4	I.a	Nopaline	Wroblewski et al. (2005); DOI: 10.1111/j.1467–7652.2005.00123.x
1D1609	Alfalfa	GCA_002943835.1	G7_a	II	Octopine	Palumbo et al. (1998); DOI: 10.1007/s002030050586
1D132	Cherry	GCA_003667725.1	G8_a	I.b	Nopaline	Wroblewski et al. (2005); DOI: 10.1111/j.1467–7652.2005.00123.x
Ach5	Yarrow	GCA_000971565.1	G1	II	Octopine	Archdeacon et al. (2000); DOI: 10.1111/j.1574–6968.2000.tb09156.x
C58	Cherry	GCA_000092025.1	G8	I.a	Nopaline	Lin and Kado (1977); DOI: 10.1139/m77-229

## Results and discussion

### *Agrobacterium* strain 1D1108 is highly efficient in stable and transient transformation

**Fig. 1.**
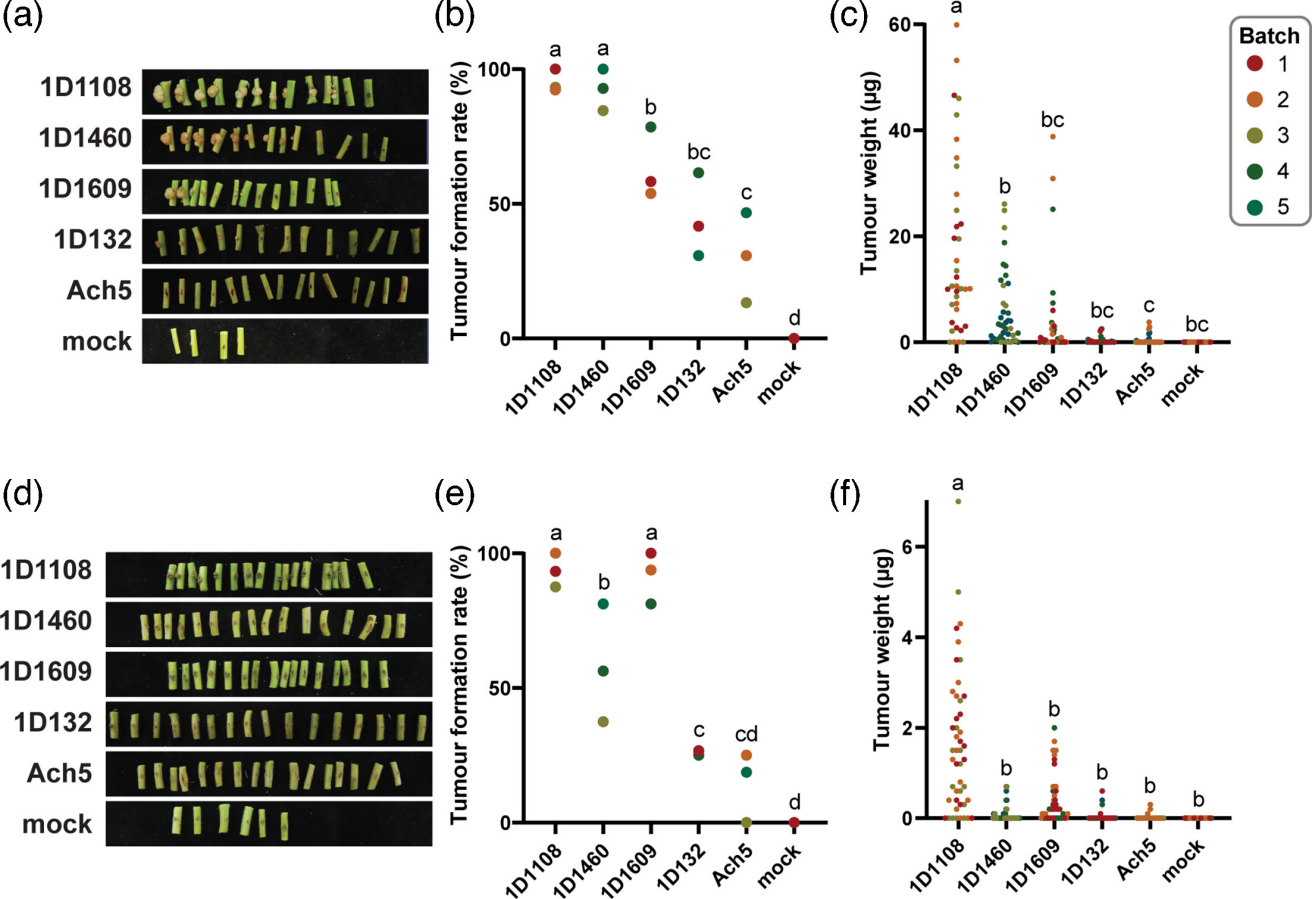
Tumour formation induced by wild-type *Agrobacterium* strains. (**a**) Representative photos, (**b**) tumour formation rates and (**c**) tumour weight distribution on kidney bean. (**d**) Representative photos, (**e**) tumour formation rates and (**f**) tumour weight distribution on soybean Tainan No. 7. The results were collected at 5 weeks after inoculation. A total of five batches, with sample sizes ranging between 11 and 16 plants per batch, were used. Each strain was tested in three different batches. The tumour formation rates among strains were compared using one-way ANOVA and Tukey post hoc test. For tumour weight, data points that deviate from the mean by more than three sd were considered as outliers and excluded.

To evaluate tumourigenesis efficiencies of the selected *Agrobacterium* strains, we measured tumour formation rates and weights at five weeks post-inoculation. The five wild-type strains examined in this work (i.e. 1D1108, 1D1460, 1D1609, 1D132 and Ach5) differed significantly in their performance against two legume hosts (Fig. 1). For inoculation on kidney bean, 1D1108 and 1D1460 both showed >90% tumour formation rates averaged across three batches, which are significantly higher than the other three strains (Fig. 1b). On soybean Tainan No. 7, 1D1108 and 1D1069 showed >90% tumour formation rates, significantly higher than the other three strains (Fig. 1e). For further comparisons, C58 was reported to have a tumour induction rate of 74.5% on kidney bean and 10.1% on soybean Tainan No. 7 [18]. For tumour weight, 1D1108 was capable of inducing significantly larger tumours than all other four strains in both hosts (Fig. 1c, f). Our quantitative assays using both metrics revealed that 1D1108 was the best-performing strain on both hosts. 1D1460 and 1D1609 also showed high virulence on both hosts, with 1D1609 as the second best on soybean and 1D1460 second to 1D1108 on kidney bean. Our assays also revealed that the induced tumours on soybean were approximately one order of magnitude smaller. Nevertheless, those induced tumours were still clearly visible upon visual inspection.

**Fig. 2.**
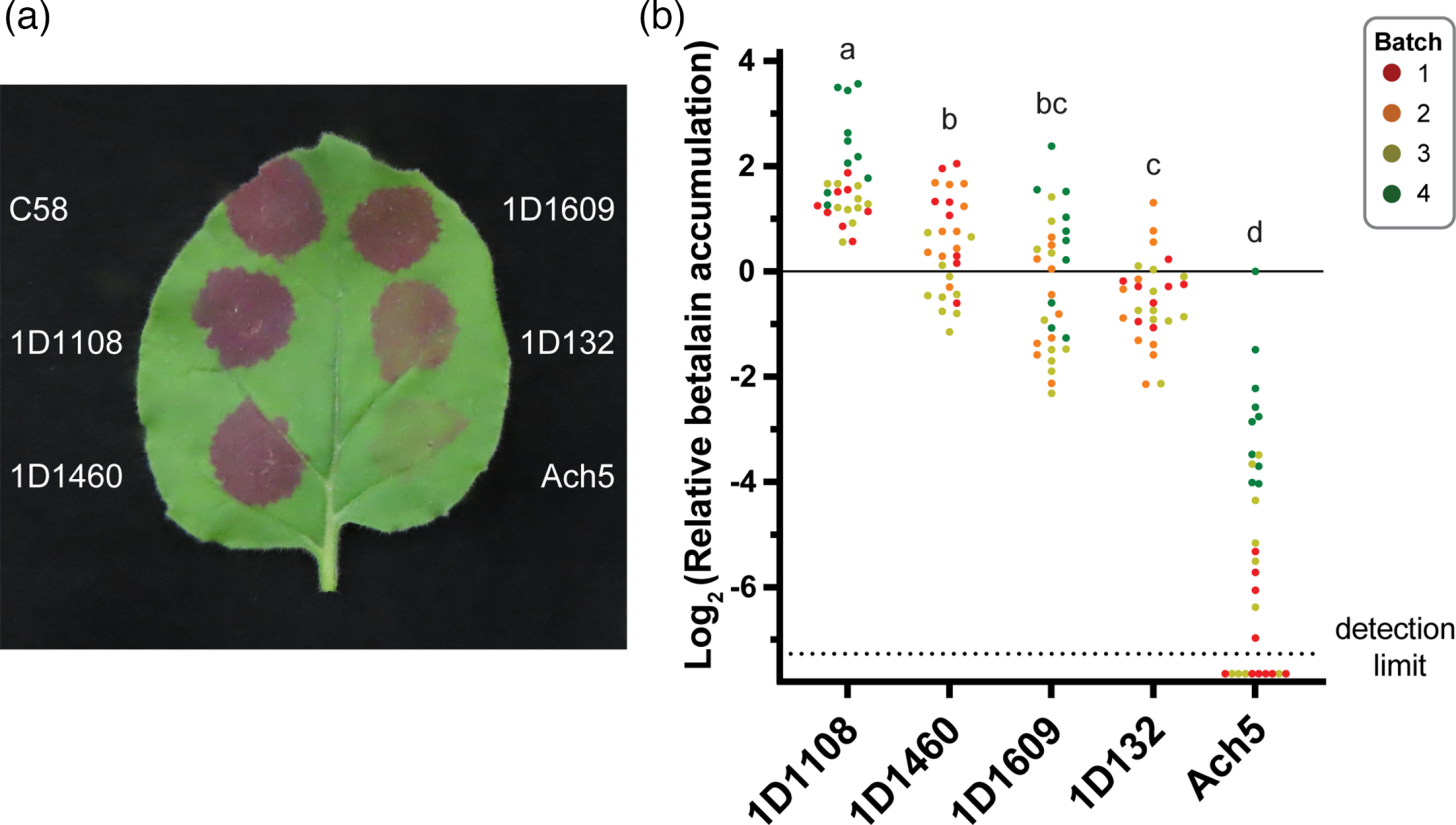
Transient transformation by wild-type *Agrobacterium* strains. (**a**) A representative photo and (**b**) relative betalain accumulation levels normalized to the reference strain C58. Leaves of 30-day-old *N. benthamiana* were used for infiltration, and results were collected at 48 hpi. A total of four batches, with sample sizes ranging between 8 and 10 leaves per batch, were used. Each wild-type strain was tested in three different batches. The reference strain C58 was infiltrated into each leaf and used for normalization. Data points with a raw betalain accumulation value of zero or below were plotted under the dotted line that indicates the detection limit. The relative betalain accumulation levels among strains were compared using one-way ANOVA and Tukey post hoc test.

For transient transformation based on agroinfiltration into mature leaves, we utilized the RUBY reporter system [29]. The expression of this reporter converts tyrosine in plant cells to the red pigment betalain, enabling precise quantification by absorbance. Our preliminary tests using kidney bean and soybean produced results that were generally weak, uneven within the infiltrated regions and variable among leaves. Therefore, *N. benthamiana* was selected for this assay. To control for variability among individual leaves and across batches, C58 was infiltrated in each leaf for normalization. Based on ~30 samples distributed across three batches for each strain, 1D1108 performed significantly better than the other four wild-type strains, averaging 3.8-fold higher signal than the reference strain C58 (Fig. 2).

In summary, our results from stable and transient transformation assays demonstrate that 1D1108, a wild-type strain that has received limited prior study, has strong potential to reveal new insights into AMT and support future improvements.

### Implementation of *in vitro* and *in planta* RNA-Seq experiments

**Fig. 3.**
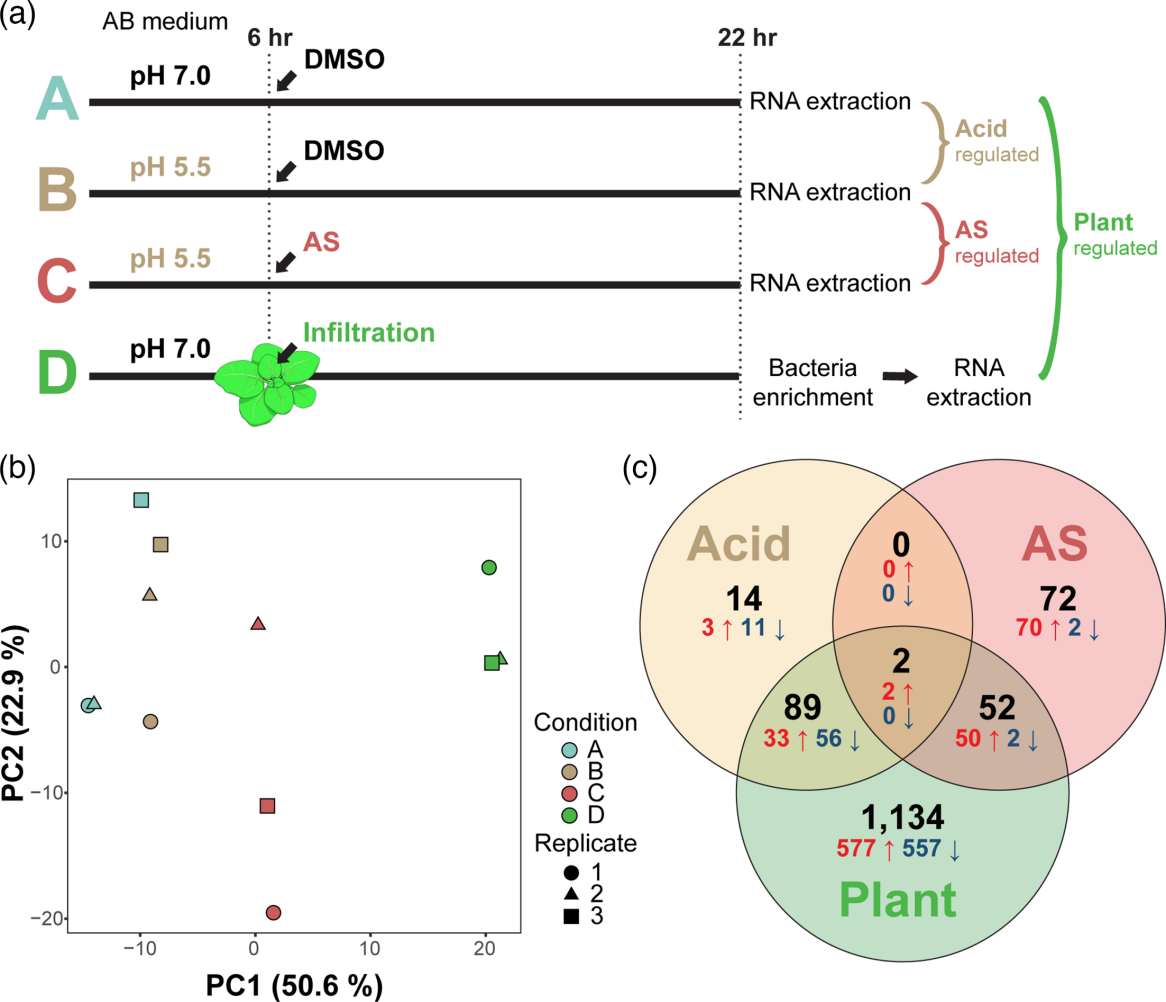
Design and summary results of the RNA-Seq experiment. (**a**) Conditions, time points and pairwise comparisons of the RNA-Seq experiment. (**b**) Principal component analysis. Dots representing individual samples are colour-coded according to the condition. The distance between dots indicates the level of dissimilarity. The percentage of variance explained by each axis is provided in parentheses. (**c**) A Venn diagram that illustrates the distribution of differentially expressed genes [DEGs; |log_2_(fold change)|≥1 and *P*-adjusted <0.01]. Among the 5,359 protein-coding genes in strain 1D1108, a total of 1,353 were differentially expressed in at least one of the pairwise comparisons between conditions. The combined numbers of DEGs were indicated in black, while the numbers of up- and down-regulated genes were indicated in red and blue, respectively.

To investigate the genes contributing to the high efficiencies of 1D1108, we conducted RNA-Seq experiments to characterize its transcriptomic responses to conditions relevant to AMT. These included four conditions, each with three biological replicates (Fig. 3a). Condition A, axenic culture in the minimal medium at pH 7.0, was used as the baseline. Conditions B and C were used to investigate the effects of two key regulators of virulence-related signal transduction, namely, an acidic environment (i.e. pH 5.5) and the presence of a phenolic compound (i.e. AS), respectively. Importantly, we established an *in planta Agrobacterium* transcriptome corresponding to the transient transformation in *N. benthamiana* in condition D. For this, we homogenized the infiltrated leaves at 16 h post-infiltration (hpi) and enriched bacterial cells via filtration and centrifugation prior to RNA extraction.

The 150 bp paired-end Illumina sequencing generated on average ~7.3 million quality-filtered reads per sample for the three *in vitro* conditions (i.e. A, B and C) (Table S1, available in the online Supplementary Material). For condition D, on average, ~38.0 million quality-filtered reads were obtained per sample. Among these, ~15.2 million reads (40%) were mapped to the genome sequence of 1D1108, and the remaining reads were almost entirely mapped to the plant genome. These results demonstrated that our bacterial enrichment process in condition D was effective. Importantly, all 12 samples exceeded the recommended sequencing depth of 2 to 3 million reads for bacterial RNA-Seq [30].

Principal component analysis revealed that differences in gene expression across conditions were primarily explained by the first principal component (PC1), which accounted for 50.6% of the total variance (Fig. 3b). In contrast, variation among biological replicates was mostly captured by PC2, explaining 22.9% of the variance. These results indicate that environmental condition was the main driver of gene expression patterns. Notably, the *in planta* transcriptome was clearly distinct from all *in vitro* conditions, highlighting extensive transcriptional reprogramming in *Agrobacterium* when colonizing plants.

### *In planta* transcriptome reveals shared and unique DEGs

Among the 5,359 protein-coding genes in strain 1D1108, 1,353 (25.2%) were identified as DEGs, defined by |log_2_(fold change)|≥1 and *P*-adjusted <0.01, in at least one of three pairwise comparisons (Fig. 3c; Table S2). The acidic environment (B vs A; ‘Acid’) resulted in 105 DEGs. Phenolic induction (C vs. B; ‘AS’) yielded a comparable number of DEGs, with 126 identified. In contrast, the plant environment (D vs. A; ‘Plant’) resulted in 1,277 DEGs.

In the plant environment, dozens of DEGs overlapped with those induced by either acidity or AS (Fig. 3c; Table S2). These included acid-induced genes encoding the type VI secretion system (T6SS) for interbacterial competition [16,31, 32], as well as AS-induced *vir* and T4SS genes involved in T-DNA transfer [17]. Notably, *Agrobacterium* cells used for *in planta* transcriptome were resuspended in infiltration buffer without AS, mimicking natural infection condition. Thus, such co-induction suggests that machinery for both competition and transformation is activated in *Agrobacterium* during plant colonization. However, 1,134 DEGs were specific to the ‘Plant’ set, underscoring the extensive regulatory responses to host-derived cues that are not elicited by acidity or AS alone.

**Fig. 4.**
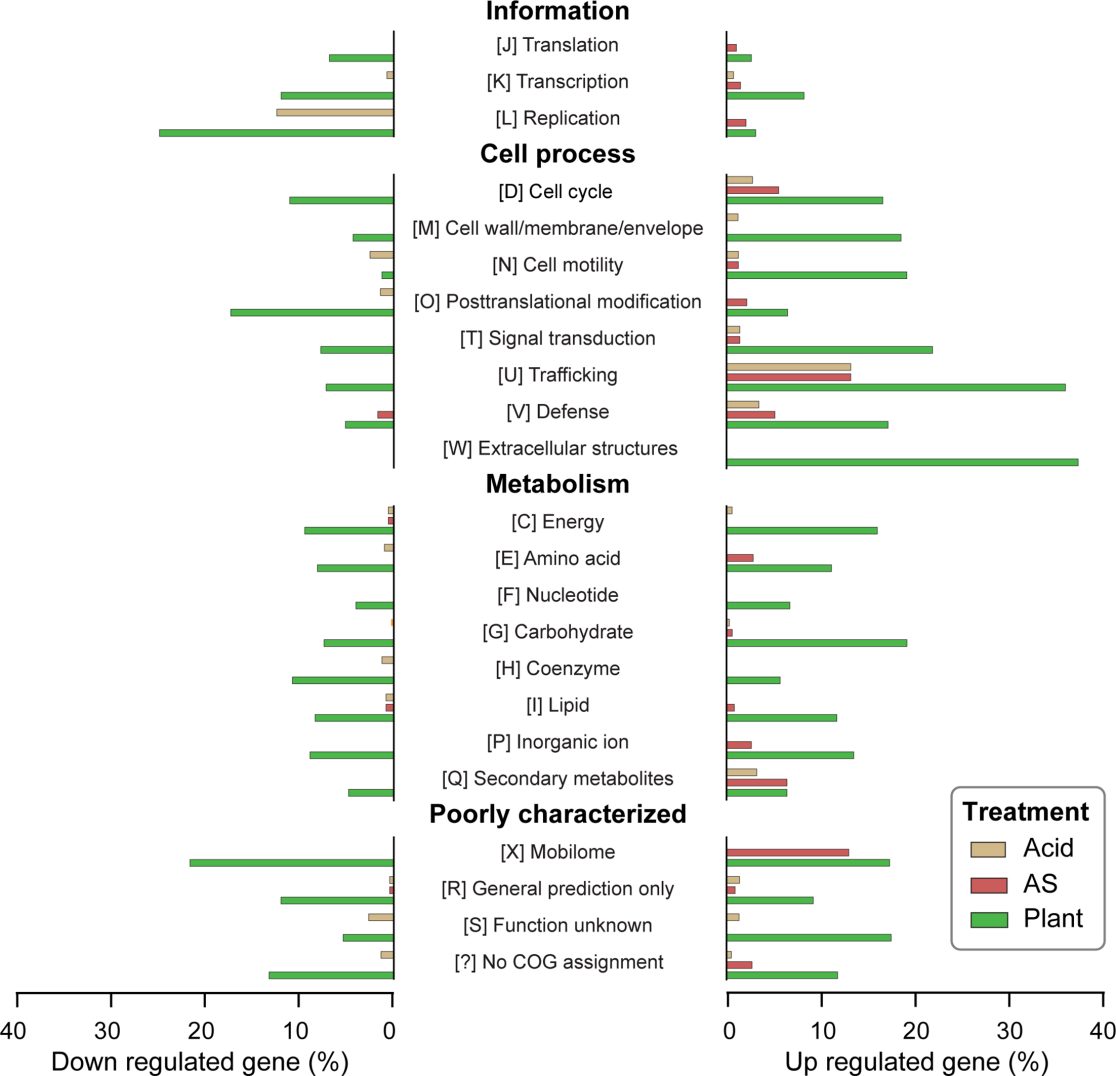
Functional category enrichment of DEGs. The functional category assignments were based on the COG. The percentages of genes that were up- and down-regulated in each functional category were plotted for the three treatments.

To explore these responses further, we conducted functional enrichment analysis using the Clusters of Orthologous Genes (COG) classification (Fig. 4; Table S2). The most enriched categories among genes up-regulated inside the plant were ‘W’ (extracellular structures, 37.5%; mainly type IV pili) and ‘U’ (intracellular trafficking, secretion and vesicular transport, 36.1%; mainly T4SS). By gene count, category ‘G’ (carbohydrate transport and metabolism) was the most represented, with 65 out of 322 genes up-regulated *in planta*. These findings illustrate the breadth of transcriptional reprogramming triggered by plant colonization.

### Distinct transcriptomic profiles among replicons

**Fig. 5.**
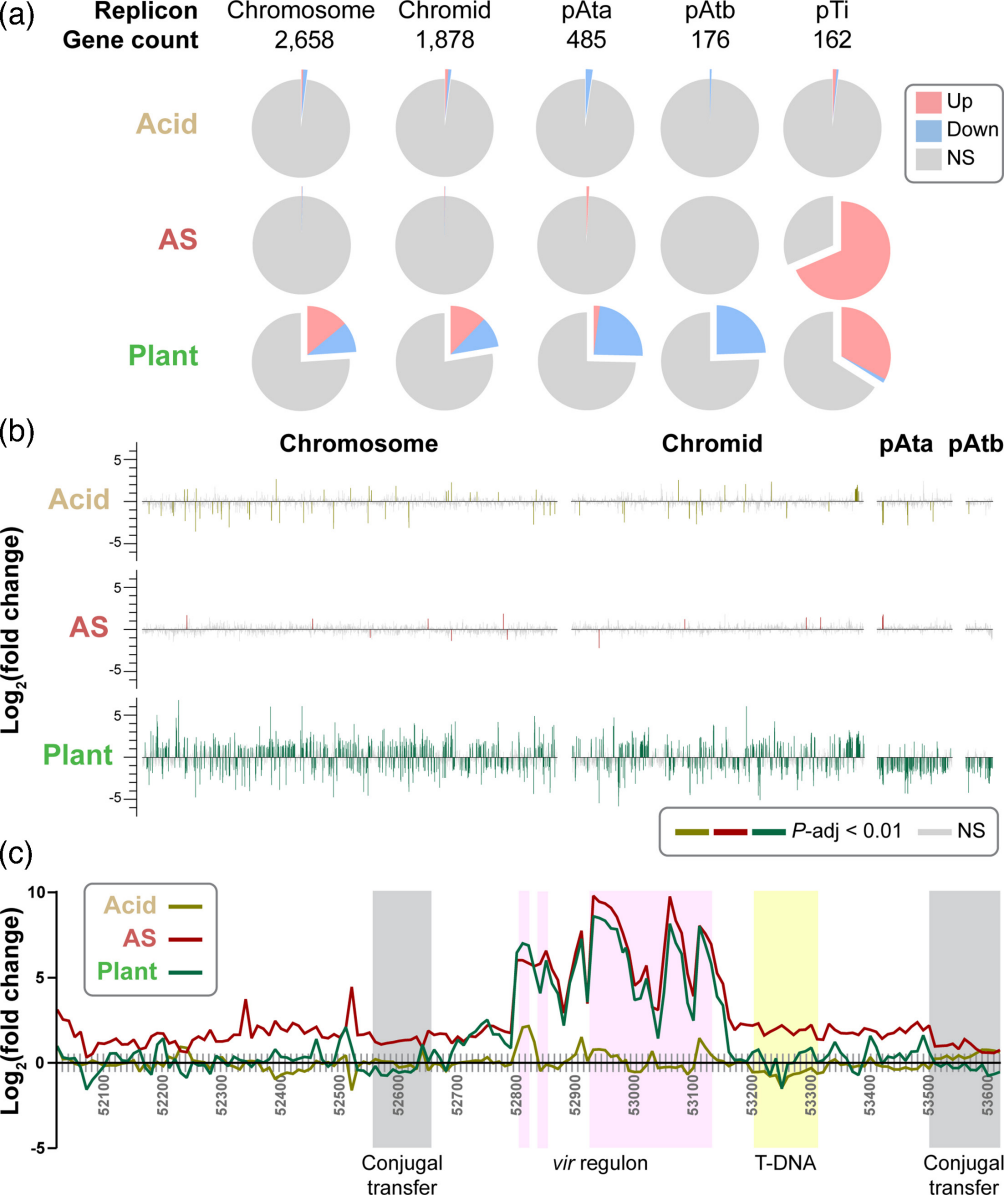
Analysis of the 1D1108 transcriptome based on genomic locations. (**a**) Distribution of DEGs [log_2_(fold change)|≥1 and *P*-adjusted <0.01] among replicons under different environmental cues. The pie charts illustrate the proportion of genes that are up- or down-regulated in each replicon under the specified factor. Abbreviation: NS, not significant. (**b**, **c**) Relative positions and expression levels of genes on each replicon. Within each replicon, all protein-coding genes were plotted in the same size and arranged based on their relative positions; non-coding regions were omitted. For panel (**c**), genes with known functional significance were shaded with background colours for highlighting (conjugal transfer: grey; *vir*: pink; T-DNA: yellow).

To assess how differential gene expression is organized at the genome level, we examined the distribution of DEGs across the replicons of 1D1108 and identified distinct patterns under the three treatments (Fig. 5a). Acidity did not result in preferential induction of DEGs across the replicons, apart from the T6SS gene cluster located near one end of the linear chromid (Fig. 5b). In contrast, AS induction led to a focused up-regulation of genes on pTi, particularly within the *vir* regulon. The plant environment triggered a more complex response: up- and down-regulated genes were observed in similar proportions on both the chromosome and chromid, while the accessory plasmids pAta and pAtb showed predominantly down-regulation. Notably, pTi exhibited strong induction under both AS induction and the plant environment, consistent with its role in virulence (Fig. 5c).

A closer examination of pTi revealed that over 20 genes in the *vir* regulon were up-regulated in response to AS induction and the plant environment (Figs5c  6; Table S2). These included regulatory genes *virA* and *virG*, along with structural and processing components such as *virB1–11*, *virD1–5* and *virE1–3*, mostly essential for T-DNA mobilization [6]. Additionally, the *accABCDEFG* operon located immediately upstream of the *vir* regulon and involved in opine transport [33] was up-regulated under acidic and plant environment.

**Fig. 6.**
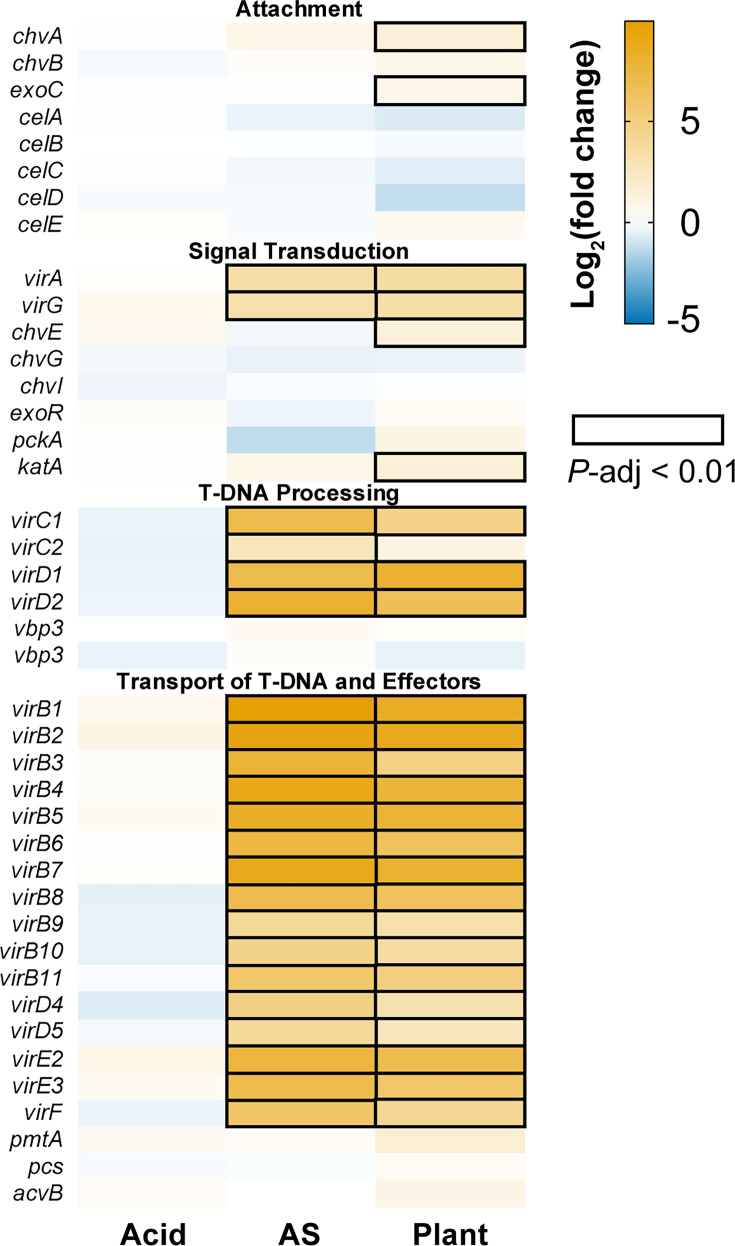
Differential expression of genes related to *Agrobacterium* virulence. The relative expression levels were plotted as a heatmap. Genes that were up- or down-regulated were plotted in orange or blue, respectively. The differential expression levels that reached statistical significance (*P*-adjusted <0.01) were indicated by black boxes.

Interestingly, only two genes, *virH1* and *virH2* encoding P450-type monooxygenases, were consistently up-regulated under all three conditions (Fig. 3c). These two genes are not essential for virulence but are likely involved in the detoxification of antimicrobial phenolic compounds, including the *vir* inducers [34].

Unexpectedly, many genes located in other regions of pTi, including those within the T-DNA, were induced by AS but not by the plant environment. This discrepancy may reflect prior observations that AS can increase pTi copy number through elevated RepABC protein expression [35]. In contrast, phenolic signals may be less abundant in the apoplast of *N. benthamiana*, or other plant-derived factors may suppress pTi replication.

While AS and plant colonization both activated *vir* genes on pTi, several relevant chromosomal genes, such as the *celABC* and *celDE* operons for cellulose fibril synthesis, did not respond to either stimulus (Fig. 6). These genes are not essential for virulence but can promote virulence by anchoring agrobacteria to plant cells [36]. Additionally, the *chvG*/*chvI* two-component system and its periplasmic repressor *exoR*, which sense acidity [31], showed constitutive expression across all conditions.

### Known and novel virulence-associated genes regulated *in planta*

In addition to the *vir* regulon genes on pTi, which were described in the previous section, we identified other known virulence-associated genes that were specifically up-regulated during plant colonization. These results further support the biological relevance of the *in planta* transcriptome. At the same time, we discovered a broad set of genes not previously associated with virulence regulation, offering new insights into *Agrobacterium*-host interaction.

Among the known factors, we observed up-regulation of *chvA* and *exoC*, chromosomal genes involved in the synthesis and export of *β*-1,2-glucan required for bacterial attachment to plant [8,9, 37] (Fig. 6). Also up-regulated were *chvE*, encoding a sugar-binding protein that modulates the VirA/VirG signal transduction system [38,39], and *katA*, encoding a catalase that detoxifies reactive oxygen species and enhances virulence [40,41].

Beyond these well-characterized genes, we identified other genes previously unknown to be regulated by plant signals and lacking evidence for their role in virulence. These include *ctpABCEF*, encoding a type IV pilus involved in reversible surface attachment [42]; *exoACHKMNOPQVY* for succinoglycan biosynthesis [43]; and *cspA*, a cold shock protein recognized as a microbe-associated molecular pattern in age-dependent plant immunity [44]. Several transport systems were also induced, including those for alpha-glucoside (*aglAEFGK*), fructose (*frcABC*), glycerol (*glpPQSTV*), phosphonate (*phnCDEH*), phosphate (*pstABCS*) and sn-glycerol-3-phosphate (*ugpABE*).

Genes down-regulated within the plant environment included those involved in cell division (*ftsEHI* and *mraZ*), thiamine biosynthesis (*thiCGO*), conjugal transfer (*traABCDFGH* and *trbBKL*) and the transport systems for alpha-1,4-digalacturonate (*aguEFG*), molybdate (*modAC*), ribose (*rbsABC*) and zinc (*znuAC*).

Together, these expression patterns demonstrate that, beyond the activation of known virulence genes, plant colonization involves broader transcriptional reprogramming. These findings reveal candidate genes potentially involved in early stages of infection and provide a foundation for future functional studies.

### Shared virulence and strain-specific gene expression in *Agrobacterium*

**Fig. 7.**
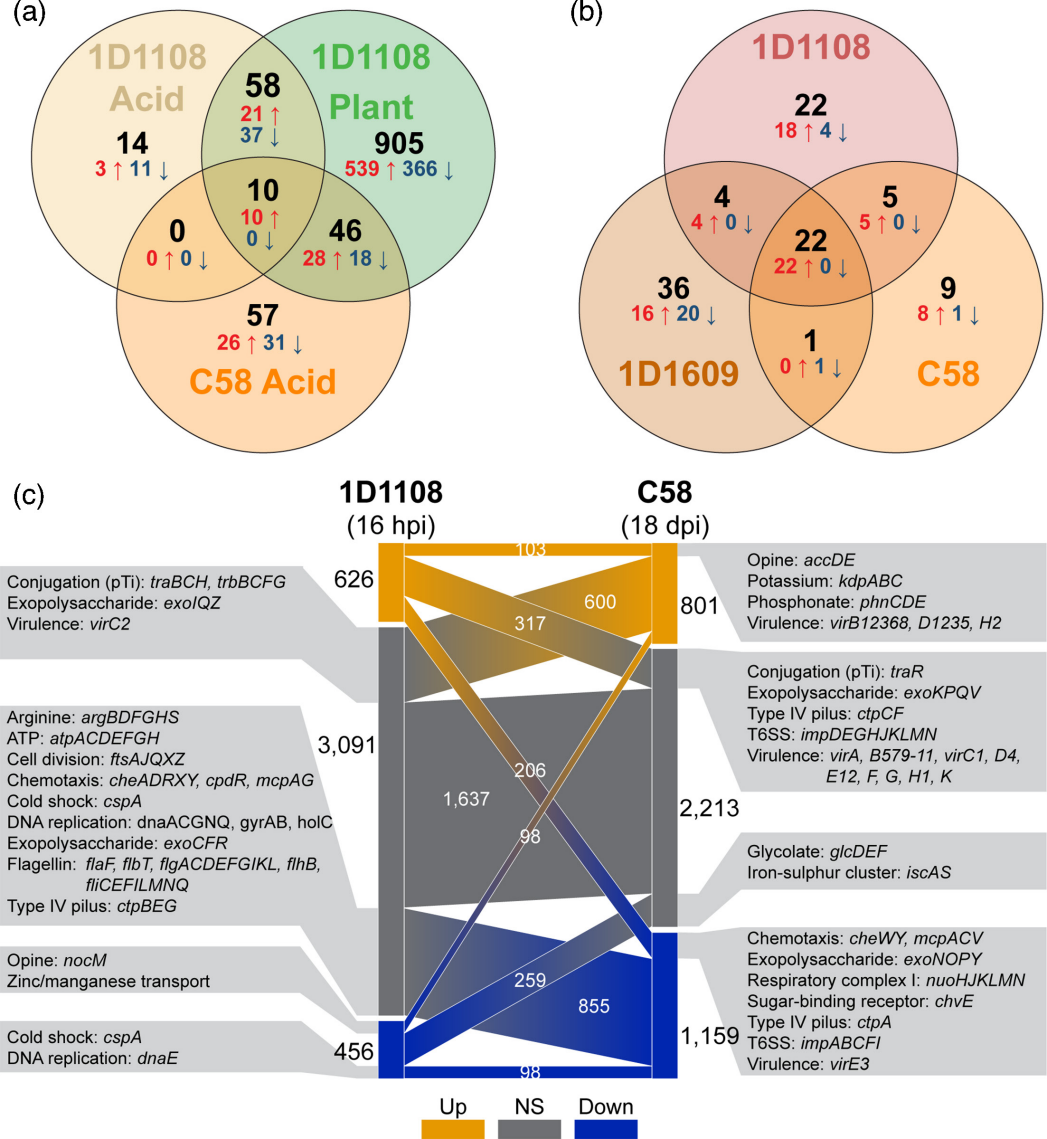
Comparisons among *Agrobacterium* transcriptome datasets. (**a**) Comparison to the acid treatment of strain C58 [19]. A total of 4,173 single-copy protein-coding genes were conserved between the two strains analysed. The combined numbers of DEGs were indicated in black, while the numbers of up- and down-regulated genes were indicated in red and blue, respectively. (**b**) Comparison to the AS treatment of strains C58 and 1D1609 [17]. A total of 3,761 single-copy protein-coding genes were conserved among the three strains analysed. (**c**) Comparison to the in-tumour transcriptome of C58 [26]. The sampling time points were 16 hpi and 18 dpi for strains 1D1108 and C58, respectively. The 4,173 single-copy protein-coding genes conserved between these two strains were separated into up-regulated (orange), no significant change (grey) and down-regulated (blue) in the two datasets. For each category, the gene count and notable genes were labelled.

To investigate the commonalities and differences in gene expression regulation among diverse *Agrobacterium* strains, we compared our RNA-Seq results to three published transcriptomics studies. First, we focused on the response to acidity. Since our *in vitro* pH 5.5 treatment was designed to mimic the plant apoplast, we included the ‘Plant’ dataset for comparison. The reference study was a microarray-based investigation of C58 grown at pH 5.5 in axenic culture [19]. Strains 1D1108 and C58 belong to different genomospecies in the same genus and have similar genome sizes, each encoding about 5,350 protein-coding genes. We identified 4,173 single-copy homologues conserved between these two strains (Table S3). Among these genes, only ten showed consistent differential expression in all three datasets (Fig. 7a), including one sulfatase, two *vir* (*virH1/H2*) and seven T6SS genes (*impBCDFGIJ*) (Table S3). This comparison revealed substantial differences in acid-responsive regulation between the strains. For example, the acidic environments induced differential expression of 72 genes in 1D1108 but not C58. These include 14 DEGs in the ‘Acid’ set and 58 DEGs in both the ‘Acid’ and the ‘Plant’ sets. Conversely, 57 genes exhibited differential expression in C58 but not 1D1108. Some of the notable DEGs in four major categories include the following: (1) up-regulated in only 1D1108: the T6SS (*impEHKLM* and *hcp*); (2) down-regulated in only 1D1108: glycine betaine/proline transport system (*proVWX*), Holliday junction DNA helicase and resolvase (*ruvABC*); (3) up-regulated in only C58: succinoglycan biosynthesis and export (*exoLTUW*); (4) down-regulated in only C58: cytochrome c oxidase (*fixOPQ*).

The second comparison examined the response to the phenolic compound AS, a known inducer of *vir* gene expression. The reference study is our previous work that used the same experimental design to investigate AS-induced expression in strains C58 and 1D1609 [17]. By including 1D1108 in the comparison, the three strains share 3,761 single-copy protein-coding genes. Only 22 DEGs were consistently up-regulated (Fig. 7b), 20 of which belonged to the *vir* regulon (Table S3). The remaining 77 DEGs showed strain-specific regulation: 67 were uniquely regulated in a single strain, and 10 shared the same direction of regulation in two strains but not all three. These genes varied widely in function and genomic location (Table S3). Together, these two cross-strain comparisons under axenic conditions highlight substantial divergence in gene regulation, even in response to shared abiotic cues.

In the third comparison, we aimed to investigate the transcriptional regulation inside host plants. However, such datasets were rare; only one suitable study was found, which investigated C58 inside *A. thaliana* tumours at 18 d post-infection (dpi) based on microarrays [26]. As observed in the two comparisons of axenic conditions, DEGs sharing the same patterns of regulation were rare (Fig. 7c). Among the 4,173 conserved homologues, only 201 showed consistent regulation, while 304 were regulated in opposite directions. Some differences may reflect species- or strain-specific regulation, but others likely result from differences in experimental design, including host plant species, tissue type and time point. Nevertheless, some general observations can be made. For example, several genes corresponding to the *vir* regulon (*virB12368*, *virD1235* and *virH2*) and opine transporter (*accDE*) were consistently up-regulated (Table S3), in line with the understanding of *Agrobacterium* ecology that these bacteria transform their hosts to obtain opines as a nutrient source [1]. Additionally, genes involved in the transport of potassium and phosphonate were consistently up-regulated, suggesting that these two substrates may be important for agrobacterial physiology inside plants.

The T6SS showed a contrasting pattern between datasets. Most T6SS genes were up-regulated in the 1D1108 in-leaf dataset but were either unchanged (*impDEGHJKLMN*) or down-regulated (*impABCFI*) in the C58 in-tumour dataset. These differences may reflect the stage of infection. At 16 hpi, the T6SS may be deployed to compete with other bacteria. In contrast, at 18 dpi, agrobacteria may already dominate the tumour microbiome, at least in small spatial scales, and repression of the T6SS activation could reduce costs. This interpretation is consistent with previous findings that the T6SS confers competitive advantages early during infection inside agroinfiltrated leaves of *N. benthamiana* within 24 hpi [16,32] but does not play a major role in shaping tumour microbiome at 60 dpi [45].

Finally, several genes associated with chemotaxis, exopolysaccharide production and type IV pilus biogenesis were up-regulated only in 1D1108 during early plant colonization, but not in the C58 in-tumour dataset. These genes may contribute to processes important at early stages of infection, such as surface sensing, attachment or migration within host tissue.

### Limited *in planta* transcriptional overlap with *P. syringae*

To assess whether plant-associated bacteria exhibit conserved transcriptomic responses during host colonization, we extended our comparative analysis to include two RNA-Seq datasets of *P. syringae* pv. *tomato* strain DC3000 [27,28]. Although *Agrobacterium* and *Pseudomonas* differ substantially in taxonomy, ecology and infection strategy, both colonize the plant apoplast and interact with host-derived cues during the early stages of infection. Therefore, a cross-species comparison provides an opportunity to assess whether common transcriptional responses are activated by plant-derived environments. Although evolutionary divergence and methodological variation pose challenges, identifying shared expression patterns could highlight convergent adaptations or core functional themes in host colonization.

**Fig. 8.**
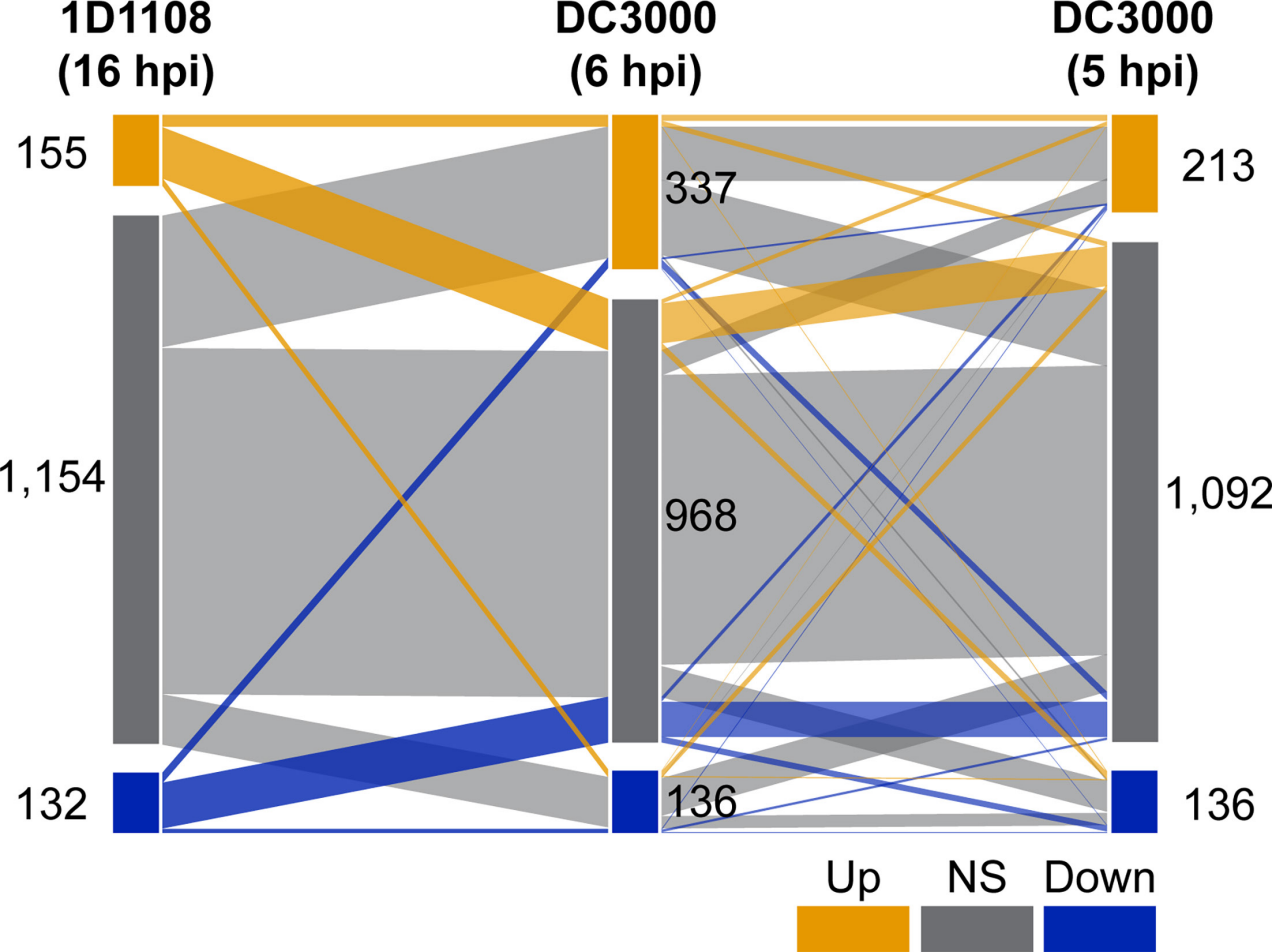
Comparisons with *in planta* transcriptomes of *Pseudomonas* strain DC3000. The *in planta* transcriptome datasets of *P. syringae* pv. *tomato* DC3000 in *A. thaliana* leaves at 6 hpi [28] and 5 hpi [27] were included for comparison. The 1,441 protein-coding genes conserved between *Agrobacterium* 1D1108 and *Pseudomonas* DC3000 were separated into up-regulated (orange), no significant expression change (grey) and down-regulated (blue). The numbers in each category and the correspondence between datasets were labelled.

For this analysis, we identified 1,441 single-copy homologues shared between 1D1108 and DC3000, accounting for approximately one-quarter of the protein-coding genes in each genome (Fig. 8; Table S3). The two DC3000 datasets included for comparison were based on infiltration into *A. thaliana* leaves, with RNA samples collected at 6 and 5 hpi, respectively. Thus, both the host plant species and the sampling time points differ from those used in the 1D1108 dataset presented in this study. Nevertheless, the same criteria of DEG inference were used.

The analysis revealed very limited congruence among the three *in planta* transcriptomes. Even between the two DC3000 datasets, which differed only slightly in the sampling time, only 135 genes were consistently up-regulated and 31 down-regulated (Table S3). When 1D1108 was included, the number of shared up-regulated DEGs dropped to 13, and only 2 genes were consistently down-regulated (Table S3). Despite the limited overlap, the shared up-regulated genes point to a coherent response involving central metabolism, nutrient uptake and stress adaptation. These included genes involved in central carbon metabolism (*accB*, *ilvI* and *sucB*), sugar utilization (*cscA* and *thuE*), amino acid and sulphur assimilation (*argJ* and *cysP*), osmoprotectant transporters (*opuBD* and *proW*) and the oxidative stress response (*ohr*). This pattern suggests that the early colonization involves metabolic reprogramming, nutrient scavenging and defences against host-derived stresses. In comparison, the two commonly down-regulated DEGs, acyl-CoA dehydrogenase (*acd*) and the accessory factor of xanthine dehydrogenase (*xdhC*), are both associated with energy metabolism. This finding suggests a suppression of purine catabolism and fatty acid degradation pathways during early infection, possibly reflecting a shift toward host-derived carbohydrates and osmolytes as primary energy sources.

Despite these shared elements, the overall low overlap, particularly between the two *Pseudomonas* datasets with similar designs, highlights substantial transcriptomic divergence across studies. Differences in host species, localized microenvironments and even slight shifts in sampling time can all contribute to these discrepancies. These results underscore the difficulty of identifying a universal *in planta* transcriptional programme across diverse study systems of bacterial strains and their hosts.

In contrast to the variability observed across studies, our own transcriptomic datasets showed high internal consistency and clear treatment-specific signatures (Fig. 3). The broader divergence among datasets likely reflects fundamental biological differences. *Agrobacterium* is a biotrophic pathogen that delivers T-DNA and effectors through its T4SS, whereas *P. syringae* employs a type III secretion system and associated effectors to suppress host immunity during hemibiotrophic infection. Despite these mechanistic differences, both systems were transcriptionally up-regulated *in planta* (Table S3), emphasizing active secretion as a shared feature of early host colonization. These findings suggest that while the transcriptional programmes deployed by different plant-associated bacteria are highly context-dependent, they converge on common functional imperatives such as resource acquisition, competition and host interface modulation.

## Conclusions

This study integrates comparative phenotyping and transcriptomic profiling to examine how environmental and host-associated cues shape gene expression in *Agrobacterium*. We focused on the wild-type strain 1D1108, which exhibited high transformation efficiencies across multiple assays. By combining *in vitro* treatments that simulate plant signals with *in planta* transcriptome analysis, we captured broad regulatory responses that influence AMT and early-stage host colonization.

Our results demonstrate that the plant environment triggers widespread and coordinated gene expression changes in *Agrobacterium*, including the activation of the *vir* regulon, secretion systems, attachment mechanisms and nutrient uptake pathways. While acidic pH and phenolic signals are established inducers of virulence, *in vitro* experiments captured only a small subset of the transcriptional dynamics observed *in planta*. These findings reveal that host-derived cues elicit a more complex and integrated response, involving metabolic reprogramming and stress adaptation that support bacterial survival and interaction with host tissue.

Cross-species comparisons at within-genus and between-class levels revealed limited overlap in transcriptional responses, underscoring the context-specific and strain-dependent nature of bacterial gene regulation. These results highlight the need to study *Agrobacterium* biology in ecologically and physiologically relevant contexts, beyond simplified axenic conditions and a few model strains.

While our results provide a useful transcriptomic resource for strain 1D1108, we acknowledge the limitation that directly linking these expression patterns to the high transformation efficiency of this strain remains challenging. To more effectively identify gene regulatory changes associated with transformation efficiency, additional comparative transcriptomics involving strains with markedly different efficiencies under identical *in planta* conditions will be required. Furthermore, integrating time-resolved sampling, multi-omics approaches and functional validation of candidate genes will be essential to unravel the molecular basis underlying strain variation, ecological adaptation and transformation efficiency.

## Methods

### Tumour assay for stable transformation efficiencies

The bacterial strains used are listed in Table 1, and the procedure was based on that described previously [18]. The strains were grown at 28 °C in 2 ml of 523 medium overnight, then diluted 10-fold and subcultured in fresh medium for 4 h. The cells were collected by centrifugation at 8,000 g for 10 min, then resuspended to OD_600_ 1.0 with 0.9% NaCl solution. The host plants, 3-week-old kidney bean (HV-120; Known-You Seed Co., Ltd., Taiwan) and soybean (Tainan No. 7; Tainan District Agricultural Research and Extension Station, Ministry of Agriculture, Taiwan), were wounded on the stem by a sterilized needle before applying 5 µl bacterial suspension. For mock inoculation, 5 µl of autoclaved 0.9% NaCl solution was used. Results were collected at 5 weeks after inoculation. The tumour formation rates were calculated based on 11 to 16 plants in three batches. For tumour weight, data points that deviate from the average by more than three sd were removed as outliers. The statistical significance was evaluated using one-way ANOVA; multiple comparisons were corrected with Tukey statistical hypothesis testing.

### Agroinfiltration for transient transformation efficiencies

The procedure was modified from an established protocol [46]. The RUBY reporter system [29], in which the transgene expression leads to a visible pigment betalain, was chosen for quantifying the transformation efficiency. Agrobacterial strains carrying the 35S:RUBY plasmid (Addgene number 160908) were grown in 3 ml of 523 medium with spectinomycin (250 µg ml^−1^) at 28 °C overnight, centrifuged at 10,000 g for 10 min, then resuspended in infiltration buffer (10 mM MES, 10 mM MgCl_2_, 150 µM AS) with OD_600_ adjusted to 0.2. Leaves of 30-day-old *N. benthamiana* were used for infiltration. For quantification, leaf discs were collected 2 days after infiltration and soaked in 200 µl betalain extraction buffer (10% EtOH, 0.1% formic acid). After incubation at room temperature overnight, 150 µl of the extraction buffer was used for absorbance measurement. The raw betalain accumulation level was calculated as ((OD_475_−OD_600_)+(OD_535_−OD_600_)). To calculate relative betalain accumulation levels, the individual measurements were normalized to values derived from the reference strain C58 obtained from the same leaf. Three batches with eight to ten leaves per batch were used for one-way ANOVA and Tukey post hoc test.

### RNA-Seq experiment

The experimental design of four conditions with three biological replicates each is illustrated in Fig. 3(a). The *in vitro* experiments were conducted based on the procedure described previously [17]. Briefly, the strains were grown on 523 agar plates at 28 °C for 3 days, and then, individual colonies were picked and grown in 5 ml of 523 liquid medium at 28 °C overnight. The bacterial cells were collected by centrifugation at 6,000 ***g*** for 4 min, then resuspended in AB-MES medium (pH 7.0) to OD_600_ 10. For each sample, 0.1 ml of the suspension was added to 4.9 ml AB-MES (pH 7.0 or pH 5.5) and cultured at 28 °C for 6 h before adding 5 µl of 200 µM AS dissolved in DMSO for induction or 5 µl of DMSO as control. For *in planta* experiments, bacterial cells in 15 ml of suspension in AB-MES (pH 7.0) were collected by centrifugation and resuspended in 15 ml of infiltration buffer (10 mM MES, 10 mM MgCl_2_) before being infiltrated into 31-day-old *N. benthamiana*. To enrich the bacterial cells, we followed the procedure described previously [28]. The infiltrated leaves were ground with liquid nitrogen in a mortar and incubated in 30 ml ice-cold bacterial isolation buffer (25 mM TCEP, 9.5% ethanol, 0.5% phenol, pH 4.5) at 4 °C with 100 r.p.m. shaking for 20 h. The buffer was filtered using 6 µm sterilized filter mesh, centrifuged at 3,200 *g* and 4 °C for 20 min before removing the supernatant. The pellet was resuspended with 900 µl ice-cold bacterial isolation buffer and centrifuged at 3,800 ***g*** and 4 °C for 20 min. After centrifugation, the upper white layer that contains enriched bacterial cells was carefully collected by pipetting into a clean 1.5 ml tube.

The RNA samples were prepared using HiYield Total RNA Extraction Kit (Cat. No. YRB50, Arrowtech, Taiwan). For each sample, the expression level of *virB1* was checked by RT-qPCR with *recA* as the internal control. The primers for these two genes are *virB1* (forward: ACCGCGCGTCCAAAAGATGAT; reverse: ACATCCCATGTTTCCTCGGAT) and *recA* (forward: AGAGAACCCGTCGAAATGGTCT; reverse: TCGGTTCCAATGAAAACGTGGTTGA). The strand-specific RNA-Seq library preparation was processed by the core facility of Academia Sinica (Taipei, Taiwan) using the TruSeq Stranded mRNA Library Prep Kit (Cat. No. 20020594, Illumina, USA). To enrich the bacterial mRNA molecules, three additional steps were incorporated. First, for the *in planta* samples, plant mRNA molecules were removed using the RNA Purification Beads from the TruSeq Stranded mRNA Library Prep Kit, which utilize poly-T oligo-attached magnetic beads to capture the poly-A tail of eukaryotic mRNA molecules. Second, for all samples, bacterial rRNA molecules were depleted using the QIAseq FastSelect −5S/16S/23S Kit (Cat. No. 335925, QIAGEN, Aarhus, Denmark). Third, for the *in planta* samples, the depletion of plant rRNA molecules was performed using the QIAseq FastSelect –rRNA Plant Kit (Cat. No. 334315, QIAGEN, Aarhus, Denmark). After these additional processing steps, all samples underwent cDNA synthesis, adapter ligation and library amplification using TruSeq Stranded mRNA Library Prep with SuperScript III Reverse Transcriptase (Cat. No. 18080044, Thermo Fisher Scientific, USA), KAPA HiFi HotStart ReadyMix (Cat. No. 07958935001/KK2602, Roche, Basel, Switzerland) and Agencourt AMPure XP (Cat. No. A63881, Beckman-Coulter, Brea, CA).

For quality control, we performed DNA quantification using Qubit dsDNA HS quantification (Cat. No. Q32854, Invitrogen, USA), Fragment Analyzer DNA size profiling using HS NGS Fragment Kit (Cat. No. DNF-474-500, Agilent Technologies, USA) and digital PCR quantification using QX200 Droplet Digital PCR EvaGreen Supermix System (Cat. No. 1864001, 1864034, Bio-Rad, USA). The Illumina paired-end sequencing based on the NovaSeq 6000 platform was outsourced to Genomics BioSci and Tech Co., Ltd. (New Taipei, Taiwan).

### Transcriptomics analysis

Unless stated otherwise, the bioinformatics tools were used with the default settings. The Illumina raw reads were processed using Trimmomatic v0.39 [47] for adaptor removal and quality trimming with the settings ‘ILLUMINACLIP:2:30:10:1:True LEADING:20 TRAILING:20 MINLEN:50’. The processed reads were mapped to *Agrobacterium* strain 1D1108 genome (GenBank accession: GCA_003666425.1) using BWA v0.7.17 [48]. The mapping results were processed using SAMtools v1.9 [49] to calculate reads in regions corresponding to rRNA, tRNA and protein-coding genes. For the *in planta* datasets, reads that did not map to the bacterial genome were mapped to the *N. benthamiana* genome (GCA_000723945.1) to confirm that those reads originated from the plant hosts, rather than contaminations.

To infer the gene expression levels, the mapped reads were counted by using HTSeq v2.0.2 [50] with the settings ‘-t CDS -s no --nonunique all’. The result was further processed using DESeq2 v1.36.0 [51] to infer the normalized expression levels based on the median-of-ratios method. The DEGs were defined based on the criteria of |log_2_(fold change)|≥1 and *P*-adjusted <0.01. To avoid spurious results, genes with total read counts <10 were excluded. Principal component analysis was performed using the ‘plotPCA’ function of DESeq2.

### Comparisons among transcriptomics studies

For comparisons with other transcriptomics studies, the genome sequences of *Agrobacterium* C58 (GCA_000092025.1), *Agrobacterium* 1D1609 (GCA_002943835.1) and *P. syringae* pathovar *tomato* DC3000 (GCA_000007805.1) were obtained from GenBank. The homologous genes among 1D1108 and other bacteria were identified using OrthoMCL v1.3 [52].

Based on the homologous genes, the lists of DEGs identified in this study were compared with those identified in previous works, including those that examined the effects of an acidic environment on C58 [19], AS treatment on strains C58 and 1D1609 [17], the in-tumour environment on C58 [26] and the in-leaf environment on DC3000 [27,28]. To maintain consistency, the lists of DEGs were all filtered based on the same criteria of |log_2_(fold change)|≥1 and *P*-adjusted (or equivalent when available) <0.01. Because the AS treatment study on C58 and 1D1609 [17] was conducted by our team, we re-analysed the raw reads using the same bioinformatic procedure as described in this study. For other comparisons, we obtained the lists of DEGs from the supplementary materials of previous publications [19,26,28].

## Usage of artificial intelligence tools

ChatGPT-4o was used to assist with brainstorming manuscript organization, suggesting improvements in wording and correcting grammar. The vast majority of the writing and the final text are the authors’ original work. No generative AI tools were used in the preparation of figures or tables.

## Supplementary material

10.1099/mgen.0.001485 Uncited Table S1.

10.1099/mgen.0.001485 Uncited Table S2.

10.1099/mgen.0.001485 Uncited Table S3.

## Abbreviations

AMT: *Agrobacterium*-mediated transformation
AS: acetosyringone
COG: Clusters of Orthologous Genes
DEGs: differentially expressed genes
dpi: days post-infection
hpi: hours post-infiltration
pRi: root-inducing plasmids
pTi: tumour-­inducing plasmids
T-DNA: transfer DNA
T4SS: type IV secretion system
T6SS: type VI secretion system
